# The Potential Use and Value of a Wearable Monitoring Bracelet for Patients With Chronic Obstructive Pulmonary Disease: Qualitative Study Investigating the Patient and Health Care Professional Perspectives

**DOI:** 10.2196/57108

**Published:** 2024-09-13

**Authors:** Suzanne M Debeij, Jiska J Aardoom, Miriam L Haaksma, Wieteke A M Stoop, Eléonore F van Dam van Isselt, Marise J Kasteleyn

**Affiliations:** 1 Department of Public Health and Primary Care Leiden University Medical Center Leiden Netherlands; 2 University Network for the Care Sector South Holland Leiden University Medical Center Leiden Netherlands; 3 National eHealth Living Lab Leiden Netherlands; 4 Department of Cardiac and Pulmonary Rehabilitation Revant Breda Netherlands

**Keywords:** eHealth, Chronic Obstructive Pulmonary Disease, COPD, wearable, exacerbation, self-management, monitoring bracelet, remote monitoring, mobile phone

## Abstract

**Background:**

The occurrence of exacerbations has major effects on the health of people with chronic obstructive pulmonary disease (COPD). Monitoring devices that measure (vital) parameters hold promise for timely identification and treatment of exacerbations. Stakeholders’ perspectives on the use of monitoring devices are of importance for the successful development and implementation of a device.

**Objective:**

This study aimed to explore the potential use and value of a wearable monitoring bracelet (MB) for patients with COPD at high risk for exacerbation. The perspectives of health care professionals as well as patients were examined, both immediately after hospitalization and over a longer period. Furthermore, potential facilitators and barriers to the use and implementation of an MB were explored.

**Methods:**

Data for this qualitative study were collected from January to April 2023. A total of 11 participants (eg, n=6 health care professionals [HCPs], 2 patients, and 3 additional patients) participated. In total, 2 semistructured focus groups were conducted via video calls; 1 with HCPs of various professional backgrounds and 1 with patients. In addition, 3 semistructured individual interviews were held with patients. The interviews and focus groups addressed attitudes, wishes, needs, as well as factors that could either support or impede the potential MB use. Data from interviews and focus groups were coded and analyzed according to the principles of the framework method.

**Results:**

HCPs and patients both predominantly emphasized the importance of an MB in terms of promptly identifying exacerbations by detecting deviations from normal (vital) parameters, and subsequently alerting users. According to HCPs, this is how an MB should support the self-management of patients. Most participants did not anticipate major differences in value and use of an MB between the short-term and the long-term periods after hospitalization. Facilitators of the potential use and implementation of an MB that participants highlighted were ease of use and some form of support for patients in using an MB and interpreting the data. HCPs as well as patients expressed concerns about potential costs as a barrier to use and implementation. Another barrier that HCPs mentioned, was the prerequisite of digital literacy for patients to be able to interpret and react to the data from an MB.

**Conclusions:**

HCPs and patients both recognize that an MB could be beneficial and valuable to patients with COPD at high risk for exacerbation, in the short as well as the long term. In particular, they perceived value in supporting self-management of patients with COPD. Stakeholders would be able to use the obtained insights in support of the effective implementation of MBs in COPD patient care, which can potentially improve health care and the overall well-being of patients with COPD.

## Introduction

Chronic obstructive pulmonary disease (COPD) is a chronic progressive inflammatory lung disease characterized by persistent respiratory symptoms and airflow limitation. It affects millions of people worldwide and places a substantial economic and health burden on societies and patients [1-3]. A major challenge associated with COPD is the occurrence and recurrence of exacerbations, which involve acute and maintained worsening of symptoms [4]. Exacerbations not only accelerate disease progression, but also lead to reduced health-related quality of life, increased health care use, poor health status, and even mortality [5-7]. It is estimated that one-third of patients with COPD who have been hospitalized due to an acute exacerbation are readmitted within 1 year [8,9]. Consequently, preventing exacerbations is an important part of COPD care. Essential to preventing exacerbations and improving health outcomes is optimizing self-management of patients with COPD [10-12]. Self-management is an iterative process during which patients use strategies to actively cope with their chronic disease within the context of their daily life [13]. Goals to optimize self-management can, for example, be directed at improving exacerbation and energy management.

In recent years, advances in telemedicine and remote patient monitoring have shown great potential to support self-management and enable timely identification and treatment of COPD exacerbations [14-20]. Wearables such as monitoring bracelets can, for example, measure various (vital) parameters to detect early COPD symptom deterioration and so allow for more timely interventions. This can reduce severity of exacerbations, and therefore, prevent hospital admissions. Besides early detection of deterioration, wearables can provide both patients and professionals with insight into patients’ recovery patterns. This may increase patients’ awareness about their health status during stable periods, before an exacerbation, and throughout the recovery period following an exacerbation. This, in turn, empowers them to engage in self-management.

While the use of a monitoring bracelet (MB) in COPD care seems promising, its integration into health care is a complex and challenging process. The monitoring device should fit the physical, social, and cultural environment [21]. Therefore, we focused on the Dutch health care setting to ensure the fit of the MB in that context. To realize this, stakeholders’ perspectives on the use of monitoring devices are of crucial importance for the successful development and implementation of such devices. Poor digital health literacy of users, security and a design that do not fit users’ needs are some of the barriers to effective implementation [22,23]. However, relatively little is known about the perspectives of health care professionals on the use of wearables to support COPD management [24]. Yet, their insights on the use and value of wearables are of crucial importance to enable successful integration into clinical practice. Regarding the patient perspective, a recent systematic review did not find a clear role of wearables in improving health outcomes in chronic disease. That is, both positive and neutral outcomes were found when investigating the impact of wearables on health care outcomes [24]. More research is needed to examine the ways in which different functionalities of wearables can aid in the management of specific types of chronic diseases. This study, therefore, aimed to explore the potential use and value of an MB for patients with COPD in the Dutch health care setting at high risk for an exacerbation. Perspectives of health care professionals (HCPs) and patients regarding the use and value of an MB in the period immediately following hospitalization and later were examined. In addition, potential facilitators and barriers to the use and implementation of a wearable were explored.

## Methods

### Study Design

This qualitative study comprised 2 semistructured focus groups, 1 with health care professionals (HCPs) and the other consisting of patients with COPD. Because of technical issues during the focus groups for patients, 3 individual interviews with patients were conducted to supplement the data. The period of data collection ran from January 31, 2023, to April 21, 2023. The reporting of this study follows the guidelines of the Consolidate Criteria for Reporting Qualitative Studies (COREQ; Multimedia Appendix 1) [25].

### Ethical Considerations

The study was declared not to fall under the scope of the Dutch Medical Research Involving Human Subjects Act (WMO) by a non-WMO review board of the Leiden University Medical Center and was granted a certificate of no objection (reference number 22-3020). The study was carried out in accordance with the General Data Protection Regulation (GDPR). All participants provided informed consent before their participation. Participants had the ability to opt out. Patients received a €25 (US $27.74) gift voucher for their participation. HCPs received a €100 (US $110.95) gift voucher. All participant data was pseudonymized to ensure confidentiality.

### Participants

Both patients with COPD and HCPs were recruited from the Revant rehabilitation center and the Franciscus Gasthuis and Vlietland hospital in the Netherlands. To be eligible to participate, patients needed to (1) be aged 18 years or older, (2) have a diagnosis of COPD, (3) have been admitted to the hospital for an acute exacerbation of COPD in the past 2 years, and (4) be able to understand, read and speak the Dutch language. The eligibility criteria for HCPs were that they must have been treating patients with COPD in one of the participating institutions. HCPs could have occupations such as pulmonary nurse, physical therapist, or pulmonologist.

### Study Procedures

Potential HCPs were purposively sampled, approached, and informed of the study by representatives from the participating institutions with an information letter. Potential patients with COPD were approached and informed of the study by their HCP. Participants were informed that Corsano Health (Corsano Health B.V.) develops medical bracelets for remote patient monitoring. Some examples of the 20 parameters that the bracelet can measure were provided. However, participants did not have the opportunity to see or try the bracelet. Before the study, participants received an invitation to join a focus group via Microsoft Teams. If patients with COPD were not able to participate in the focus group, they were scheduled for an individual interview by phone.

### Focus Groups and Interviews

A total of 4 female researchers participated (authors JA, SD, and MK, and researcher EO) in the data collection. Participants had not met the researchers before and did not have knowledge of the interviewer. All researchers had experience in qualitative research and made notes. No other people were present during focus groups and interviews. The focus groups and individual interviews both followed a predetermined semistructured interview guide (Multimedia Appendix 2). At the start of the interview, descriptive characteristics were collected. The main part of the interviews and focus groups contained questions regarding the participants’ attitudes, wishes, and needs for an MB, as well as potential facilitators and barriers related to the use and implementation of an MB. The questions covered the recovery period directly after an exacerbation as well as long-term use in the prevention of exacerbations. To gather participants’ perspectives on all these topics, they were presented with an example of an existing MB (ie, the Corsano Cardiowatch [Corsano Health B.V.]) and its corresponding functionalities were explained. The Corsano Cardiowatch is a medically certified health MB that is able to continuously measure health-related parameters such as heart rate, heart rate variability, breathing rates, skin temperature, physical activity (eg, steps and cadence), and sleep. These parameters are measured by an accelerometer and photoplethysmogram sensor. The bracelet offers flexible data collection intervals, and data are subsequently privately stored in a health cloud. These data can be safely and continuously transferred to the patient’s health care professional through a digital platform. Hence, the watch is an unobtrusive tool to continuously monitor a patient’s (vital) parameters. An example of a question asked is “What are your general needs and ideas about the monitoring bracelet?”

### Data Analyses

Descriptive analyses were conducted to summarize the sociodemographic characteristics of patients and HCPs. The interview was audio recorded and the recording was stored in a restricted secure data folder of the Leiden University Medical Centre. Interviews were transcribed and not returned to participants. Due to technical issues, one recording failed and so notes made during this interview were used in the data analysis. The transcribed interviews were coded and analyzed according to the principles of the framework method [26]. Data were coded using Atlas.ti software (version 22, ATLAS.ti Scientific Software Development GmBH). Coding was performed by 2 researchers (SD and JA or MK). The second coder repeated the process on samples from the interviews to verify codes. A code tree was created, and codes were discussed until consensus was reached. The analysis was used to categorize recurrent and common themes and to identify key elements in the interviews. Participants did not provide feedback on the findings.

## Results

### Overview

A total of 5 patients with COPD and 6 HCPs participated in this study. The HCPs worked in a hospital or in a rehabilitation center as a pulmonologist (n=2), nurse (n=2), nurse practitioner (n=1), or physical therapist (n=1). Patients were aged between 60 and 80 years and had COPD GOLD (Global Initiative for Chronic Obstructive Lung Disease) stage 3 or 4. The interviews took approximately 30 minutes and the focus groups took 1 hour. Results are presented as (1) MB in support of self-management, (2) expected use of an MB during the course of illness, and (3) facilitators and barriers. The exact number of individuals that were invited for participation and declined is unknown.

### MB in Support of Self-Management

The first aspect that most patients described as valuable in an MB is its ability to help with the (very) early recognition of an exacerbation. Some participants specifically stated that it should register and interpret trends in (vital) parameters, and subsequently notify them in case of deviations from normal values.

…*I expect the bracelet to warn me that there is something wrong with me before I notice it myself.* [male, patient]

Valuable (vital) parameters that an MB should be able to measure, according to participants, are oxygen saturation, respiration (frequency), heart rate, and physical activity, because these measures are informative about a person’s health status. For physical activity, different variables were mentioned such as: step count, type of performed activities (such as standing or walking), and pace of movement. One HCP felt that step count is not an accurate parameter for early detection of exacerbations and emphasized the greater importance of vital parameters. Also mentioned by 1 HCP was the importance of additional information about a patient’s perspective by offering the option of self-monitoring in an MB.

Another potential benefit frequently mentioned by both patients and HCPs was receiving alerts when data deviate from normal. Both groups acknowledged the importance of HCPs having access to their patients’ monitoring data. However, there was no consensus on who should receive these alerts first. Some patients thought that the HCP should be alerted first and subsequently reach out to the patients, while other participants thought it was the patients’ responsibility to reach out if something is wrong.

…*When the time comes, you can call for help yourself. The watch doesn't have to do that, I can do it myself. The most important thing is that the watch lets me know me when something is wrong.* [female, patient]

Most HCPs further mentioned that an MB should support patients in having ownership over their health. An MB can foster this ownership and give patients more autonomy by providing body awareness, finding rest, and providing awareness of and insight into normal values and trends. The HCPs further reported possible benefits for patients in terms of being able to ask for more specific help with their COPD when parameters deviate from normal, building confidence, reducing anxiety, feeling heard, and supporting the transition from the rehabilitation center to their home. HCPs could for example discuss unusual data with patients and explore the reasons behind such deviations.

…*I can imagine people will be motivated as they gain insight into their movement patterns and whether these stay within the set norms. That they become more confident and are able to ask for more specific help when they see deviations.* [HCP]

### Benefits and Drawbacks of a Monitoring Bracelet

Benefits mentioned by patients were being able to act quickly, a sense of reassurance, heightened body awareness, improved insight into the disease and normal values and patterns, and finally, improved insight for informal caregivers into how the patient is doing. Possible drawbacks were also mentioned. Most HCPs felt that some patients might just focus on the data and not think critically, become obsessed with the data, or feel that their privacy was being invaded. They also described that the MB system should be integrated with the electronic patient dossier, because using multiple systems at the same time is laborious. Some patients also described the potential drawback of becoming obsessed with data, invasion of their privacy, and reluctance due to having to use yet another application. Several patients did not see any drawbacks in using an MB.

### The Expected Use of Monitoring Bracelet During the Course of Illness

Most participants did not anticipate any potential difference between the short-term and the long-term use of an MB. Several participants said that an MB should be worn through all phases, that is, periods of well-being, exacerbation, and recovery, to obtain a comprehensive view of a patient’s condition. It is important to remember that during recovery, parameters may still deviate from normal. One thing highlighted by HCPs that might be of added value in the long-term is a parameter to measure physical activity. HCPs did not consider step count to be suitable for detecting exacerbations in the short-term, but a decrease in the trend of steps taken could be a sign of deteriorating health in the long-term. Patients did not differentiate between the importance of parameters in the long-term or the short-term.

HCPs further stated that patients could try the MB in the health care setting, to see if it suits their abilities and needs. An MB could support the transition from a care setting to the home setting, by giving insight into changes in parameters.

…*It might help us to better guide patients' transition from 10 weeks of comprehensive care to a “go do it yourself at home” approach. What difficulties do patients encounter? We often hear back: “it just didn't work out” or “I failed again” and then [with an MB] we can point out more specifically “I notice that...”* [HCP]

### Facilitators and Barriers

A major facilitator for the use of an MB is providing support to patients on how to use the device. Some HCPs thought that patients will not be able to use an MB correctly without such support.

…*I think this bracelet would be useless without instruction. Then* [with instructions] *you will know if you’re going into the right direction or maybe in a wrong direction.* [HCP]

The support could be in the form of a manual, video instructions, or even a whole support system with coaching from HCPs or support groups with other patients. This system could include coaching appointments with HCPs for regular feedback on parameters. Patients elaborated on the crucial role of support with statements about a decrease in motivation if they did not know how to use an MB. One patient mentioned that it would be useful if informal caregivers could also receive information about an MB.

HCPs also indicated that it was their responsibility to assess whether an MB could be of value to a person, and to provide guidance and coaching accordingly. Several HCPs said that patients in rehabilitation could learn how to use, practice, and interpret the system. Patients need to gain experience with an MB to know what values are normal within their own physique.

…*I think it is our role to coach the patient. So giving good instructions, adequate cut-off scores, like this is when you need to get in touch and if you're within this range you are okay.* [HCP]

In addition, an MB should be easy to use, should not malfunction, should provide signals that are tailored to a patient, and it should have an attractive design. Patients also described that it should have additional features compared with a regular smartwatch. Instructions on how to use and interpret the obtained data are an important precondition for MB use. Another important precondition for the use of an MB is ensuring the accuracy of the data and the reliable detection of abnormal values and trends. Additional preconditions mentioned by participants included standard procedures and protocols, an adequate battery life of the MB, ensuring privacy, affordable cost, and the bracelet being waterproof.

Despite a generally positive attitude, HCPs also see some barriers to the use of an MB in health care. For example, they mentioned the time required to monitor the data, which could lead to an increased demand for staff and therefore increased costs. They further mentioned that patients with COPD are often older and of lower socioeconomic status, which are risk factors for lower digital literacy. Patients described some of the same barriers, such as the potential costs, while also identifying some barriers on a more practical level, such as having to wear the device all the time. Furthermore, 1 patient mentioned she was afraid she would be overlooked if an MB malfunctioned.

…*They* [doctors] *are all so incredibly busy. I’m afraid I will be overlooked if I wear the watch, and something is wrong with the watch. We're already being overlooked now.* [female, patient]

## Discussion

### Principal Findings

The results of this study show that Dutch patients and Dutch HCPs both have an overall positive and comparable view of the use and value of an MB in the care of patients with COPD. The most frequently mentioned value of an MB for patients with COPD, according to both HCPs and patients, is its support in self-management of COPD. In addition, HCPs indicated that an MB should support patients’ ownership of their health. Furthermore, the support that patients would need to use an MB as well as the support that an MB could provide, were discussed. The potential benefits most frequently highlighted by HCPs and patients related to early detection and alerting in case of exacerbation. There was no consensus on whether the alerts should be received by the HCP or patient first. Participants did not anticipate major differences regarding the expected use of an MB during the course of illness. The most frequently mentioned facilitators for using an MB were adequate support (eg, from HCP or peers) and instructions on how to use it correctly, ease of use, and an appealing design. Potential barriers were the need for time and staff to guide patients, costs (eg, for purchase or guidance), and having to use multiple apps at the same time.

The need for support described by both HCPs and patients in this study in using and interpreting data from an MB is in line with the literature focused on patients. Target groups that have more problems with understanding and accessing eHealth in particular, are known to need more support in using it [27]. Previous literature shows that a platform supporting self-management of people with COPD was used more when participants received more personal support in how to use it [28]. HCPs in this study highlighted the importance of a comprehensive support system. This is in contrast with earlier literature where HCPs primarily emphasized the necessity of technological support alone [29]. Although a patient’s motivation influences the use of eHealth, support increases the effectiveness of and adherence to eHealth interventions [30]. Support from others should be managed carefully, as factors such as the bond between patient and the person giving support and perceived legitimacy of the person giving support can influence the use of an eHealth intervention [27,30].

Participants also described the support that an MB could provide at various time points in COPD, including stable phases and during recovery from an exacerbation. Support during these phases can be provided in various domains, which Gardener et al [31] grouped into 4 overarching categories: physical, psychological, social, and spiritual. Participants described support in the physical domain in terms of self-management. This form of support fits into the “managing symptoms and medications” domain of support for managing life with COPD [29,31]. The support that an MB can provide in managing one’s COPD, was also highlighted by participants in the study of Wu et al [32], who conducted a qualitative study on the needs of patients with COPD in applications that support their self-management. Other studies that implemented eHealth interventions and tools to support self-management of long-term conditions suggest that eHealth is safe and best used alongside usual care [33]. eHealth is most promising in blended care settings [28,34]. As factors such as personality, lifestyle, progression of a chronic disease, and environmental characteristics influence self-management, they should be taken into consideration when developing eHealth to support self-management.

In addition, the ability to accurately detect early signs of an exacerbation by measuring deviations in (vital) parameters was highlighted as the most important characteristic an MB should have both shortly after an exacerbation as over a longer period. This is not surprising, as exacerbations have a detrimental effect on health and having an exacerbation is a major risk factor for another exacerbation [35]. Other predictors of exacerbations include comorbidities, disease severity, symptom burden, and environmental factors [36,37]. Predictors such as these cannot be measured by an MB. Nevertheless, other predictors of an exacerbation, such as decreased physical activity, pulse rate, oxygen saturation, and respiratory rate, can be measured by an MB [36-39]. If an exacerbation is recognized and treated in time, the length and severity of the exacerbation can be reduced, which can support patients’ short- and long-term recovery [40].

An MB should be easy to use, according to the participants. This is in line with recent literature which suggests that simple eHealth interventions are more likely to be feasible and effective [34]. The desire of the elderly target group for an uncomplicated and familiar design of eHealth has been described earlier [41]. Therefore, an MB should be adapted to the skills, wishes, and needs of the target population [42]. The target population for an MB in COPD management does not only consist of patients, but also of HCPs. However, there is a lack of understanding regarding the perspective of the HCPs on the use of MBs in COPD care [24]. This study suggests that Dutch HCPs hold a positive view that complements the patient perspective. The inclusion of this additional perspective is of upmost importance, since cocreation with the target population can provide insight into the skills, wishes, and needs and thus facilitate an MBs use [41]. In addition, known barriers for target groups should be considered, such as cognitive barriers, physical disabilities, and lack of motivation [23,41]. To ensure an optimal fit for the target population in a national context, not only the patient but also other stakeholders should be involved in the development process [43,44].

As such, the use of nonobtrusive wearable devices appears to be beneficial and well accepted by patients and HCPs to be integrated into existing care, with little difference between long-term and short-term care. An MB could be combined with other eHealth solutions, such as smartphone apps and air quality monitoring devices to increase the impact. To effectively implement an MB supporting the management of COPD, HCPs, patients, developers, and other stakeholders in the Dutch context should be informed and involved in the process. In both long-term and short-term care, an MB could help improve overall well-being, health outcomes, and quality of life of patients with COPD.

### Limitations

Participants indicated they were not familiar with the possibilities of an MB, which suggests that they may not be aware of all the facilitators and barriers relating to the use and implementation of an MB. For example, because they were not able to see or try the MB, they could not experience the unobtrusive, small, lightweight design, and long battery life. Another limitation of this study is the small sample size. As a result, we are not sure if data saturation is sufficient to provide a representative perspective of the population. Nonetheless, given the explorative nature of this study, the sample size was sufficient to provide a first insight into the potential value of an MB. Furthermore, it may have been too difficult for patients with low digital literacy to participate in interviews via video calls, which may have led to selection bias and reduced generalizability.

### Conclusions

This qualitative study showed that both HCPs and patients recognize that an MB could be beneficial and valuable for patients with COPD at high risk of exacerbation, both in the short-term and long-term. In particular, they highlighted the perceived value of an MB in supporting the self-management of patients with COPD. Both HCPs and patients wanted to use an MB for the early detection of exacerbations and did not see major differences in its use in short-term or long-term care. Furthermore, the identified facilitators and barriers to the use and implementation of an MB in COPD care, emphasize the need for ongoing research and careful consideration of stakeholders’ perspectives. This may ultimately support the successful implementation and adoption of wearable technology in this area of care. The integration of an MB into clinical practice through blended care has the potential to increase overall well-being and health outcomes of patients with COPD by supporting their self-management.

## Appendix

Multimedia Appendix 1 COREQ (Consolidate Criteria for Reporting Qualitative Studies) checklist.

Multimedia Appendix 2 Interview protocols.

## Abbreviations

COPD: chronic obstructive pulmonary disease
COREQ: Consolidate Criteria for Reporting Qualitative Studies
GDPR: General Data Protection Regulation
GOLD: Global Initiative for Chronic Obstructive Lung Disease
HCP: health care professional
MB: monitoring bracelet
WMO: Dutch Medical Research Involving Human Subjects Act

## Data Availability

The data are not readily available because participants did not give consent to share data with other parties.
