# Supplementary material for: The Potential Use and Value of a Wearable Monitoring Bracelet for Patients With Chronic Obstructive Pulmonary Disease: Qualitative Study Investigating the Patient and Health Care Professional Perspectives
Source: JMIR Form Res. 2024 Sep 13;8:e57108. doi: 10.2196/57108 (PMC11437227; doi:10.2196/57108)
Supplement: Multimedia Appendix 2 [file formative_v8i1e57108_app2.pdf]

Appendix 2: Interview protocols

Appendix 2.1 Dutch - Interview protocol voor de focusgroepen met COPD patiënten

Versie 1, 2-6-2022

1. Introductie

Welkom en introductie gespreksleider en andere aanwezigen

a. Inleiding: introductie + CardioWatch (hierna te noemen: armband) en overzicht focusgroep-onderwerpen
b. Huisregels
c. Deelnamevergoeding
d. Praktische vragen?

2. Voorstelrondje deelnemers

Elke deelnemer stelt zich kort voor: leeftijd, ernst COPD (mild, matig, ernstig), aantal jaren COPD sinds diagnose, andere chronische ziekten.

3. Hoofdvragen

Hoofdvraag 1: U bent allen onlangs opgenomen geweest met een longaanval COPD. Hoe ging het met u in de **periode voor de longaanval**?

1.1 Voelde u aankomen dat u een longaanval kreeg? Zo ja, waaraan voelde u dat?
1.2 Denkt u dat u een longaanval kan voorkomen? Zo ja, wat doet u zelf om een longaanval te voorkomen?
1.3 Wat zou u (mogelijk) kunnen helpen om een longaanval te voorkomen in de toekomst?

Hoofdvraag 2: Wat zijn uw algemene ideeën over de armband en wat zijn uw behoeften?

2.1 Zou u de armband dagelijkse willen dragen? Waarom wel/niet? Welke factoren dragen daar mogelijk aan bij?
2.2 Wat voor ondersteuning is er volgens u (mogelijk) nodig om de armband goed te kunnen gebruiken in de behandeling voor COPD? U kunt hie*r* bijvoorbeeld denken aan ondersteuning in de vorm van informatie of ondersteuning van uw zorgverlener.
2.3 Wat zijn volgens u mogelijke factoren die het gebruik van de armband makkelijker maken?
2.4 En wat zijn volgens u mogelijke factoren die het gebruik van de armband moeilijker maken?

Hoofdvraag 3: Wat zou de rol van een armband kunnen zijn in de **periode na ontslag uit het ziekenhuis** na een longaanval?

2.1 Hoe denkt u dat de armband kan helpen in uw herstel?
2.2 Hoe zou de armband kunnen helpen om een (snelle) heropname in het ziekenhuis te voorkomen?

2.3 Welke gezondheidsparameters, dus waarden die iets zeggen over uw gezondheid zijn hierbij (mogelijk) van belang? Denk bijvoorbeeld aan waarden zoals hartslag, aantal stappen, slaap of het zuurstofgehalte in het bloed.
2.4 Verwacht u dat bepaalde van deze waarden u zouden kunnen waarschuwen voor een longaanval, voordat deze daadwerkelijk plaatsvindt?
2.5 Hoe ziet u de rol van zorgverleners bij het inzetten van de armband? Wat zou u van uw zorgverleners verwachte, welke wensen zou u hebben?

Hoofdvraag 4: Wat zou de rol van een armband kunnen zijn in het **voorkomen** van een longaanval (op lange termijn)?

3.1 Hoe denkt u dat de armband kan helpen om op langere termijn longaanvallen te voorkomen? Is dit anders dan op de korte termijn?
3.2 Welke gezondheidsparameters, dus waarden die iets zeggen over uw gezondheid zijn hierbij (mogelijk) van belang? Denk bijvoorbeeld aan waarden zoals hartslag, aantal stappen, slaap of het zuurstofgehalte in het bloed.
3.3 Verwacht u dat bepaalde van deze waarden u zouden kunnen waarschuwen voor een longaanval, voordat deze daadwerkelijk plaatsvindt?
3.4 Zou de armband u kunnen helpen om in het dagelijks leven om te gaan met uw COPD? Zo ja, hoe / op wat voor manier?
3.5 Hoe zou een eventueel ondersteuningsprogramma waarbij gebruik gemaakt wordt van de armband eruit kunnen zien? Welke functies zouden nuttig / wenselijk zijn?

Hoofdvraag 5: Wat zijn de mogelijke bedoelde en onbedoelde effecten van het gebruik van de armband?

5.1 Wat zijn voor u mogelijke voordelen van het gebruik van de armband?
5.2 Wat zijn voor u mogelijke nadelen van het gebruik van de armband?
5.3 Wat ziet u als mogelijke voor- en nadelen wat betreft het contact en uw relatie met uw zorgverlener?

4. Afsluiting

a. Van al de dingen die we vandaag besproken hebben, wat vond u het belangrijkst?
b. Heeft u nog aanvullende opmerkingen? Zijn er bepaalde zaken nog niet besproken?
c. Einde / afsluiting focusgroep

Appendix 2.2 English - Interview protocol for focus group with COPD patients

English translation from *Interview protocol voor de focusgroepen met COPD patiënten (Versie 1, 2-6-2022)*

1. Introduction

Welcome and introduction of the moderator and other attendees

e. Introduction: introduction + CardioWatch (hereafter referred to as: bracelet) and overview focus group topics
f. House rules
g. Participation compensation
h. Practical questions?

2. Introduction participants

Each participant briefly introduces themselves: age, severity of COPD (mild, moderate, severe), number of years of COPD since diagnosis, other chronic diseases.

3. Main questions

Main question 1: You have all recently been hospitalized with a lung attack COPD. How were you doing in the **period before the lung attack**?

1.4 Did you feel that you were having a lung attack? If so, how did you feel that?
1.5 Do you think you can prevent a lung attack? If so, what do you do yourself to prevent a lung attack?
1.6 What could (possibly) help you to prevent a lung attack in the future?

Main question 2: What are your general ideas about the bracelet and what are your needs?

2.5 Would you like to wear the bracelet daily? Why or why not? What factors could possibly contribute to this?
2.6 What kind of support do you think is (possibly) needed to be able to use the bracelet properly in the management of COPD? For example, you can think of support in the form of information or support from your healthcare provider.
2.7 What factors do you believe might make using the bracelet easier?
2.8 And what factors do you think might make using the bracelet more difficult?

Main question 3: What could be the role of a bracelet in the **period after discharge from the hospital** after a lung attack?

2.6 How do you think the bracelet can help in your recovery?
2.7 How could the bracelet help to prevent a (quick) readmission to the hospital?
2.8 Which health parameters, i.e., values that say something about your health, are (possibly) important here? Think, for example, of values such as heart rate, number of steps, sleep, or the oxygen level in the blood.
2.9 Do you think that some of these values could alert you to a lung attack before it actually happens?

2.10 How do you see the role of caregivers in using the bracelet? What would you expect from your caregivers, what wishes would you have?

Main question 4: What could be the role of a bracelet in **preventing** a lung attack (long-term)?

3.6 How do you think the bracelet can help prevent lung attacks in the longer term? Is this different from the short term?
3.7 Which health parameters, i.e., values that say something about your health, are (possibly) important here? Think, for example, of values such as heart rate, number of steps, sleep, or the oxygen level in the blood.
3.8 Do you think that some of these values could alert you of a lung attack before it actually happens?
3.9 Could the bracelet help you deal with your COPD in everyday life? If so, how/in which way?
3.10 How could a possible support programme that makes use of the bracelet look like? What features would be useful/desirable?

Main question 5: What are the possible intended and unintended effects of using the bracelet?

5.4 What are the possible advantages of using the bracelet for you?
5.5 What are the possible disadvantages of using the bracelet for you?
5.6 What do you see as possible advantages and disadvantages in terms of contact and your relationship with your healthcare provider?

4. Closing

d. Of all the things we have discussed today, what did you think was the most important?
e. Do you have any additional comments? Are there certain issues that have not yet been discussed?
f. End/Closing of Focus Group

Appendix 2.3 Dutch - Interview protocol voor de focusgroep met zorgverleners

Versie 1, 2-6-2022

1. Introductie

Welkom en introductie gespreksleider en andere aanwezigen

i. Inleiding: introductie + CardioWatch (hierna te noemen: armband) en overzicht focusgroep-onderwerpen
j. Huisregels
k. Deelnamevergoeding
l. Praktische vragen?

2. Voorstelrondje deelnemers

Elke deelnemer stelt zich kort voor: leeftijd, functie/type zorgverlener, en aantal jaren ervaring met behandeling COPD patiënten.

3. Hoofdvragen

Hoofdvraag 1: Wat zou de rol van een armband kunnen zijn in de **periode na ontslag uit het ziekenhuis** na een longaanval?

1.1 Zou de armband kunnen helpen in het herstel van patiënten? Zo ja: hoe?
1.2 Zou de armband kunnen helpen om een (snelle) heropname van patiënten te voorkomen? Zo ja: hoe?
1.3 Welke gezondheidsparameters (bijvoorbeeld hartslag, aantal stappen, slaap of saturatie (Zuurstof-gehalte in het bloed) zijn hierbij (mogelijk) van belang?
1.4 Verwacht u dat bepaalde van deze parameters kunnen dienen als zogenaamde "vroegtijdige waar-schuwingen"?
1.5 Hoe ziet u de rol van zorgverleners bij het inzetten van de armband? Welk verwachtingen of be-hoeften/wensen heeft u?

Hoofdvraag 2: Wat zou de rol van een armband kunnen zijn in het **voorkomen** van een longaanval (op lange termijn)?

2.1 Zou de armband kunnen helpen om op langere termijn longaanvallen te voorkomen? Zo ja: hoe? Is dit anders dan op de korte termijn?
2.2 Welke gezondheidsparameters (bijvoorbeeld hartslag, aantal stappen, slaap of saturatie (Zuurstof-gehalte in het bloed) zijn hierbij (mogelijk) van belang?
2.3 Verwacht u dat bepaalde van deze parameters kunnen dienen als zogenaamde "vroegtijdige waar-schuwingen?
2.4 Hoe ziet u uw rol als zorgverlener bij het inzetten van de armband? Welk verwachtingen of be-hoeften/wensen heeft u?

2.5 Hoe zou een interventie waarbij gebruikt gemaakt wordt van de armband eruit kunnen zien?

Hoofdvraag 3: Wat zijn uw algemene ideeën over de armband en wat zijn uw behoeften?

4.1 Zou u de armband willen inzetten in de behandeling van COPD patiënten? Waarom wel/niet? Welke factoren dragen daar mogelijk aan bij?
4.2 Wat is er nodig om de armband goed te kunnen gebruiken in de behandeling van patiënten met COPD?
4.3 Wat zijn volgens u mogelijke factoren die het gebruik van de armband binnen de behandeling die u biedt zouden faciliteren?
4.4 En wat zijn volgens u eventuele belemmerende factoren?

Hoofdvraag 4: Wat zijn de mogelijke bedoelde en onbedoelde effecten van het gebruik van de armband?

2.6 Wat zijn voor u mogelijke voordelen van het gebruik van de armband binnen de behandeling van COPD patiënten?
2.7 Wat zijn voor u mogelijke nadelen van het gebruik van de armband?
2.8 Wat ziet u als mogelijke voor- en nadelen wat betreft uw relatie met uw patiënten?

Hoofdvraag 5: In hoeverre komt de huidige doelgroep COPD patiënten (binnen het laatste jaar opgenomen geweest door een longaanval) overeen met de COPD patiënten die u ziet in de praktijk?

4. Afsluiting

g. Van al de dingen die we vandaag besproken hebben, wat vond u het belangrijkst?
h. Heeft u nog aanvullende opmerkingen? Zijn er bepaalde zaken nog niet besproken?
i. Einde / afsluiting focusgroep

Appendix 2.4 English - Interview protocol for the focus group with healthcare providers

English translation from *Interview protocol voor de focusgroep met zorgverleners (Versie 1, 2-6-2022)*

1. Introduction

Welcome and introduction to the moderator and other attendees

m. Introduction: introduction + CardioWatch (hereafter referred to as: bracelet) and overview focus group topics
n. House rules
o. Participation compensation
p. Practical questions?

2. Introduction participants

Each participant briefly introduces themselves: age, position/type of healthcare provider, and number of years of experience with treatment of COPD patients.

3. Main questions

Main question 1: What could be the role of a bracelet in the **period after discharge from the hospital** after a lung attack?

1.6 Could the bracelet help in the recovery of patients? If so, how?
1.7 Could the bracelet help to prevent (rapid) readmission of patients? If so, how?
1.8 Which health parameters (e.g., heart rate, number of steps, sleep, or saturation (oxygen level in the blood) are (possibly) important here?
1.9 Do you expect that some of these parameters can serve as so-called "early warnings"?
1.10 How do you see the role of caregivers in using the bracelet? What expectations or needs/desires do you have?

Main question 2: What could be the role of a bracelet in **preventing** a lung attack (long-term)?

2.9 Could the bracelet help to prevent lung attacks in the longer term? If so, how? Is this different from the short-term?
2.10 Which health parameters (e.g., heart rate, number of steps, sleep, or saturation (oxygen level in the blood) are (possibly) important here?
2.11 Do you expect that some of these parameters can serve as so-called "early warnings"?
2.12 How do you see your role as caregiver in applying the bracelet in COPD management? What expectations or needs/desires do you have?
2.13 How could an intervention using the bracelet look like?

Main question 3: What are your general ideas about the bracelet and what are your needs?

4.5 Would you like to use the bracelet in the treatment of COPD patients? Why or why not? What factors could possibly contribute to this?

4.6 What is needed to effectively use the bracelet in the treatment of patients with COPD?
4.7 What do you think are possible factors that would facilitate the use of the bracelet within the treatment you provide?
4.8 And what do you think are barriers?

Main question 4: What are the possible intended and unintended effects of the use of the bracelet?

2.14 What are the possible advantages of using the bracelet in the treatment of COPD patients?
2.15 What are the possible disadvantages of using the bracelet for you?
2.16 What do you see as potential advantages and disadvantages in terms of your relationship with your patients?

Main question 5: To what extent does the current target group of COPD patients (admitted to hospital due to a lung attack within the last year) correspond to the COPD patients you see in practice?

4. Closing

j. Of all the things we have discussed today, what did you think was the most important?
k. Do you have any additional comments? Are there certain issues that have not yet been discussed?
l. End/Closing of Focus Group
